# Efficacy and safety of proprotein convertase subtilisin kexin type (PCSK9) inhibitors in patients with acute coronary syndrome: A systematic review and meta-analysis

**DOI:** 10.1097/MD.0000000000038360

**Published:** 2024-05-31

**Authors:** Ruohong Song, Jinsong Li, Yan Xiong, Hui Huang, Xiaojian Liu, Qiyong Li

**Affiliations:** a Department of Cardiology, Sichuan Tianfu New District People’s Hospital, Chengdu, China; b Department of Cardiology, Sichuan Provincial People’s Hospital, Chengdu, China.

**Keywords:** ACS, effective, meta-analysis, PCSK9

## Abstract

**Background:**

The effect of proprotein convertase subtilisin kexin type (PCSK9) inhibitors on blood lipids and major adverse cardiovascular events (MACEs) is still controversial for acute coronary syndrome (ACS) patients. This study aimed to evaluate the efficacy and safety of PCSK9 inhibitors for ACS patients.

**Methods:**

We searched the following databases until March 2023: PubMed, Embase, Cochrane, Web of Science, CNKI, Chongqing VIP Database and Wan Fang Database. Finally, all randomized controlled trials, retrospective studies and prospective studies were included in the analysis.

**Results:**

A total of 20 studies involving 48,621 patients were included in this meta-analysis. The results demonstrated that PCSK9 inhibitors group was more beneficial for ACS patients compared to control group (receiving statins alone or placebo). The meta-analysis showed: there was no significant difference in high density lipoprotein cholesterol between PCSK9 inhibitors group and control group (standard mean difference = 0.17, 95% confidence interval [CI]: −0.02 to 0.36, *P* = .08), while the level of low density lipoprotein cholesterol in PCSK9 inhibitors group was lower than that in control group (standard mean difference = −2.32, 95% CI: −2.81 to −1.83, *P* < .00001). Compared with the control group, the PCSK9 inhibitors group also decreased the levels of total cholesterol and triglycerides (mean difference = −1.24, 95% CI: −1.40 to −1.09, *P* < .00001, mean difference = −0.36, 95% CI: −0.56 to −0.16, *P* = .0004). Moreover, compared with the control group, PCSK9 inhibitors group could reduce the incidence of MACEs (relative risk [RR] = 0.87, 95% CI: 0.83–0.91; *P* < .00001). However, this study showed that the incidence of drug-induced adverse events in PCSK9 inhibitors group was higher than that in the control group (RR = 1.15, 95% CI: 1.05–1.25, *P* < .0001).

**Conclusion:**

Although this study demonstrates that PCSK9 inhibitors have higher drug-induced adverse events, they can not only reduce low-density lipoprotein cholesterol levels but also reduce the incidence of MACEs simultaneously. However, these findings needed to be further verified through large sample, multicenter, double-blind randomized controlled trials.

## 1. Introduction

The prevalence and mortality of acute coronary syndrome (ACS) are on the rise worldwide.^[1]^ Cardiovascular disease is linked to a range of risk factors, with dyslipidemia, particularly high levels of low density lipoprotein cholesterol (LDL-C), standing out as a significant contributor.^[2]^ Hence, reducing the level of LDL-C is a crucial step in preventing cardiovascular issues and enhancing the outcomes for individuals with heart conditions.^[3]^ Statins reduce the incidence of major adverse cardiovascular event (MACE) by lowering the LDL-C levels.^[4]^ Nevertheless, studies have indicated that approximately 30% of statin users may experience muscle-related symptoms, while 0.5% to 2% patients may encounter liver complications.^[5]^ Consequently, there is a pressing need for novel lipid-lowering medications that are both safer and more efficacious in enhancing the prognosis for ACS patients.

Proprotein convertase subtilisin kexin type (PCSK9) inhibitors represent a novel class of lipid-regulating drugs that have emerged in recent years, targeting cholesterol metabolism.^[6]^ Statins can reduce the LDL-C in the blood by 20% to 50%, but PCSK9 inhibitors have shown the ability to reduce LDL-C levels by an average of 60%.^[7,8]^ While numerous studies have confirmed the lipid-lowering benefits of PCSK9 inhibitors, there remains a paucity of evidence regarding their effectiveness in managing blood lipid profiles and cardiovascular outcomes in ACS patients. Hence, we undertook a systematic review and meta-analysis to assess the existing data on the efficacy and safety of PCSK9 inhibitors specifically in individuals with ACS.

## 2. Materials and methods

### 2.1. Study design

This systematic review and meta-analysis adhere to the Preferred Reporting Items for Systematic Reviews and Meta-Analysis statement guidelines.^[9]^

### 2.2. Literature retrieval strategy

Two researchers (Y.X. and R.S.) independently conducted literature retrieval, and in cases of disagreement, a third researcher (Q.L.) participated in discussions to finalize the retrieval strategy. The following electronic databases: PubMed, Embase, the Cochrane, Web of Science, CNKI, Chongqing VIP Database and Wan Fang, were searched up to March 2023. All randomized controlled trials (RCTs), prospective cohort studies, and retrospective studies were included. The retrieval method adopts the combination of subject words and free words (the following search terms were used: (“Acute coronary syndrome”) AND (“PCSK9 inhibitor”OR “evolocumab”OR “alirocumab” OR“Proprotein convertase subtilisin type 9”OR “Proprotein convertase kexin type 9”). Specific search strategies for each individual database are provided in the supplementary materials.

### 2.3. Inclusion and exclusion criteria

#### 2.3.1. Inclusion criteria

We utilized the Population, Intervention, Comparators, Outcomes, Study Design framework to identify relevant studies Population, Intervention, Comparators, Outcomes, Study Design^[10]^: P: patients were diagnosed with ACS, regardless of whether they underwent percutaneous transluminal coronary intervention (PCI). I: PCSK9 inhibitors were used. C: placebo or statins were used. O: outcomes measures encompassing LDL-C, HDL-C, TC, TG, MACEs, and drug-induced adverse events. MACEs include cardiac death, myocardial infarction, fatal or nonfatal ischemic stroke, angina, and so on. Drug-induced adverse events include injection-site reaction, muscle-related event, rhabdomyolysis, neurocognitive disorder, hepatic disorder and so on. S: RCT, retrospective study and prospective cohort study.

### 2.4. Exclusion criteria

Exclusion criteria: inability to extract relevant data; studies not in Chinese or English; non-journal publications and duplicate reports; other drugs were used; animal studies, case reports, letters, reviews, withdrawn studies and meta-analysis.

### 2.5. Data extraction

According to the inclusion and exclusion criteria, 2 researchers (Y.X. and R.S.) independently reviewed the full text: Data extraction encompassed: basic information such as authors, year of publication, intervention group, control group, outcomes, and study duration; study design type encompassed RCTs, prospective cohorts, and retrospective studies. In cases where essential clinical information was missing, attempts were made to contact the corresponding author for clarification.

### 2.6. Risk of bias assessment and grading quality of evidence assessment

The retrospective studies were assessed by using Newcastle–Ottawa Scale (NOS), and studies scoring ≥7 were considered to be of high quality. The NOS consists of 8 items, categorized into 3 dimensions including selection, comparability, and depending on the study type, either outcome (for cohort studies) or exposure (for case-control studies). Additionally, RCTs were evaluated using the Cochrane risk of bias tool. The items on the list were divided into 7 domains: generation of the allocation sequence; concealment of the allocation sequence; blinding; attrition and exclusions; other sources of bias; biases specific to trial design; and biases that might be specific to a clinical specialty. Bias assessments were independently conducted by 2 researchers (H.H. and J.L.). Any unresolved disagreements between the 2 reviewers were resolved through discussion or reviewed by a third reviewer (Q.L. and X.L.).

### 2.7. Statistical analysis

All analyses were performed using comprehensive meta-analysis statistical software (RevMan version 5.3; Cochrane Collaboration, Oxford, United Kingdom). The dichotomous outcomes were reported by relative risk (RR), and continuous outcomes were reported for mean difference (MD) and SMD. Heterogeneity test was performed by Chi-square test. If *P* ≥ .1 and *I*^2^ ≤ 50%, it was judged to indicate that there was no heterogeneity, and a fixed-effect model was used. If *P* < .1 or *I*^2^ > 50%, it was judged indicate that there was a high heterogeneity, and a random effect model was used. We also performed a sensitivity analysis to identify the source of the heterogeneity, by eliminating the included literature one by one.

## 3. Result

### 3.1. Search result

**Figure 1.**
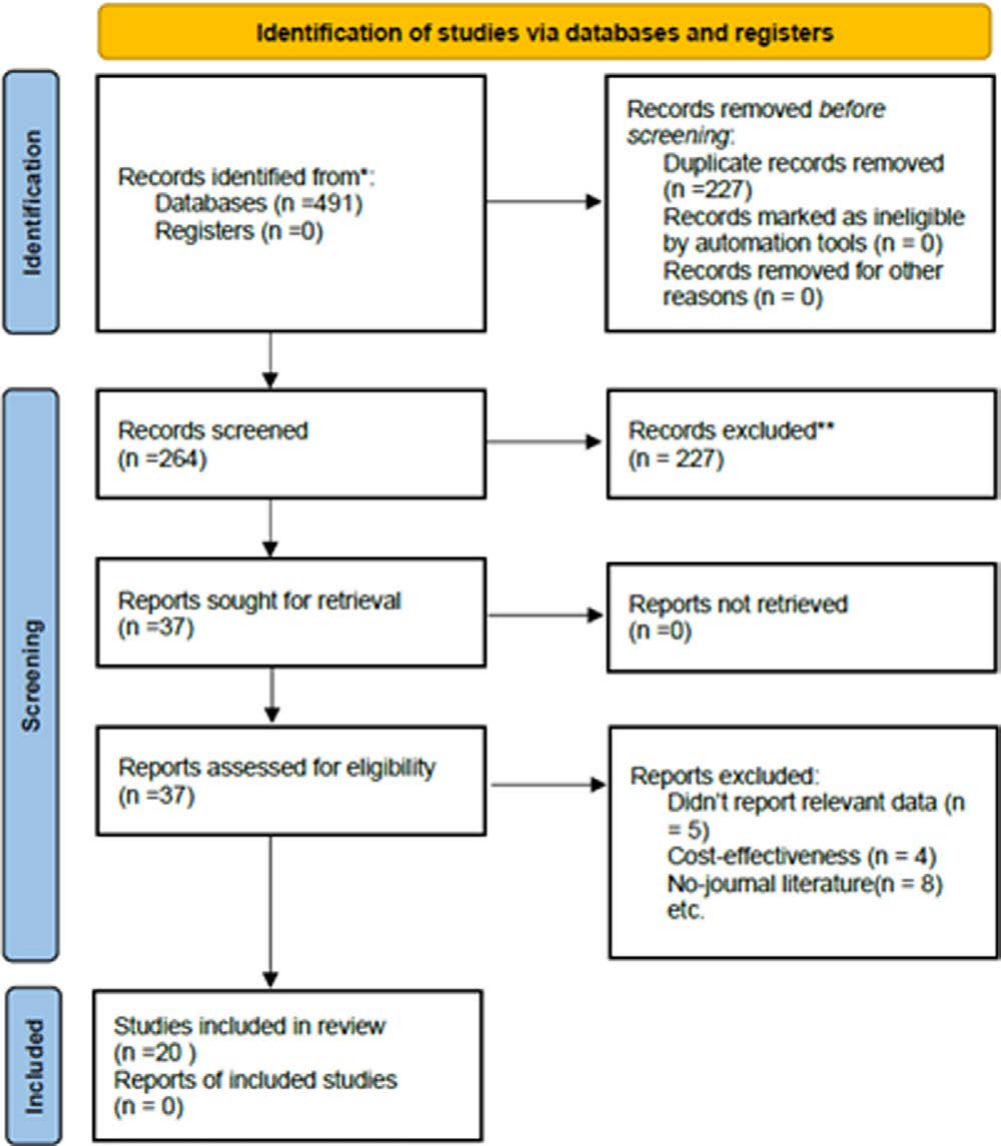
Flow diagram.

The initial search yielded 491 records, of which PubMed included 68 records, Embase included 186 records, Cochrane included 117 records, CNKI included 91 records, Wan Fang Database included 20 records, Chongqing VIP Database included 4 records, while the remaining 5 records were obtained through other sources. Of these results, we excluded 227 due to the duplication. After reading the titles, abstracts, and full texts, 20 potentially eligible studies were assessed for inclusion criteria. Eleven studies published in English, and 9 studies published in Chinese were included in this meta-analysis. Figure 1 displays the selection algorithm, and numbers of included and excluded studies.

### 3.2. Study characteristics

Thirteen studies were RCTs,^[11–23]^ 4 studies were retrospective cohort studies,^[24–27]^ and 3 studies were prospective cohort studies.^[28–30]^ A total of 20 studies included 48,621 patients. Among those included studies, the main basic characteristics of the included literatures are shown in Table 1.

**Table 1:** Basic characteristics of the included literature.

Name	Year	Age (I/C)	Study type	Number of persons (I/C)	Intervention group	Controlled group	Inclusion criteria	Follow-up time (I/C)
Lin et al^[11]^	2021	65.49/65.93	RCT	80/80	PCSK9 I (420 mg every month + atorvastatin (20 mg qn)	Atorvastatin (20 mg qn)	PCI	3 mo/3 mo
Feng et al^[12]^	2020	64.25/63.78	RCT	50/50	PCSK9 I (140 mg q2w) + atorvastatin (20 mg qd)	Atorvastatin (80/20 mg qd)	PCI	1 mo/1 mo
Huang et al^[13]^	2020	61.4/62.0	RCT	30/30	PCSK9 I (140 mg q2w)	Atorvastatin (20 mg qd)	NA	3 mo/3 mo
Lei et al^[24]^	2021	59.51/62.26	R	41/87	PCSK9 I (140 mg q2w) + atorvastatin (20 mg qn)	Atorvastatin (20 mg qn)	PCI	6 mo/6 mo
Tao et al^[25]^	2021	62.14/61.64	R	57/52	PCSK9 I (140 mg q2w) + atorvastatin (20 mg qd)	Atorvastatin (20 mg qd)	PCI	8 wk/8 wk
Wang et al^[14]^	2022	59.3/56.1	RCT	42/42	PCSK9 I (140 mg q2w) + atorvastatin (20 mg qn) or rosuvastatin (10 mg qn)	Atorvastatin (20 mg qn) or rosuvastatin (10 mg qn)	NA	3 mo/3 mo
Wang et al^[26]^	2021	54.97/54.16	R	124/50	PCSK9 I (140 mg q2w) + atorvastatin (20 mg qd) or rosuvastatin (10 mg qd)	Atorvastatin (20 mg qd) or rosuvastatin (10 mg qd)	NA	12 wk/12 wk
Ye et al^[28]^	2021	65.2/62.0	P	11/12	PCSK9 I (75 mg q2w) + atorvastatin (20 mg qd) or rosuvastatin (10 mg qd)	Atorvastatin (20 mg qd) or rosuvastatin (10 mg qd)	PCI	3 mo/3 mo
Zhao et al^[15]^	2021	60.3/58.7	RCT	44/50	PCSK9 I (140 mg q2w) + atorvastatin (20 mg qn)	Atorvastatin (20 mg qn)	PCI/CABG	6 mo/6 mo
Gao et al^[16]^	2021	61.3/61.3	RCT	30/31	PCSK9 I (75 mg q2w) + atorvastatin (20 mg qd) or rosuvastatin (10 mg qd)	Atorvastatin (20 mg qd) or rosuvastatin (10 mg qd)	NA	36 wk/36 wk
Ako et al^[17]^	2019	61.8/60.5	RCT	93/89	PCSK9 I (75 mg q2w) + atorvastatin (≥10 mg qd) or rosuvastatin (≥5 mg qd)	Atorvastatin (≥10 mg qd) or rosuvastatin (≥5 mg qd)	NA	36 wk/36 wk
Koskinas et al^[18]^	2019	60.5/61	RCT	155/152	PCSK9 I (420 mg every month) + atorvastatin (40 mg/80 mg qd)	Atorvastatin (40 mg/80 mg qd)	PCI/CABG	8 wk/8wk
Li et al^[29]^	2021	60.6/58.6	P	54/45	PCSK9 I (140 mg q2w) + rosuvastatin (10 mg qn)	Rosuvastatin (10 mg qn)	NA	8 wk/8 wk
Schwartz et al^[19]^	2018	58/58	RCT	9462/9462	PCSK9 I (75 mg q2w) + atorvastatin (40 mg/80 mg qd) or rosuvastatin (20 mg/40 mg qd)	Placebo (q2w)	NA	48 mo/48 mo
Trankle et al^[20]^	2019	59	RCT	10/10	PCSK9 I (150 mg 1 mo) + atorvastatin (40 mg/80 mg qd) or rosuvastatin (20 mg/40 mg qd)	Placebo	NA	14 d/14 d
Xu et al^[30]^	2021	56.53/58.91	P	96/238	PCSK9 I (140 mg q2w) + atorvastatin (20 mg qd) or rosuvastatin (10 mg qd)	Atorvastatin (20 mg qd) or rosuvastatin (10 mg qd)	PCI/CABG	12 wk/12 wk
Yano et al^[27]^	2020	64.6/65.2	R	18/40	PCSK9 I (140 mg q2w) + rosuvastatin (5 mg qd)	Rosuvastatin (5 mg qd)	PCI	12 wk/12 wk
Leucker et al^[21]^	2021	53/53	RCT	30/27	PCSK9 I (420 mg 1 mo) + high intensity statins	Placebo	NA	30 d/30 d
Sabatine et al^[22]^	2018	62.5/62.5	RCT	13,784/13,780	PCSK9 I (140 mg q2w) + high intensity statins	Placebo + high intensity statins	NA	2.2 yr/2.2 yr
Meng et al^[23]^	2022	58/58	RCT	30/30	PCSK9 I (140 mg q2w) + atorvastatin (40 mg/d)	Atorvastatin (40 mg/d)	NA	NA

NA = not available, P = prospective cohort study, PCI = percutaneous coronary intervention, R = retrospective study, RCT = randomized controlled trial.

### 3.3. The bias risk assessment results of the included studies

**Figure 2.**
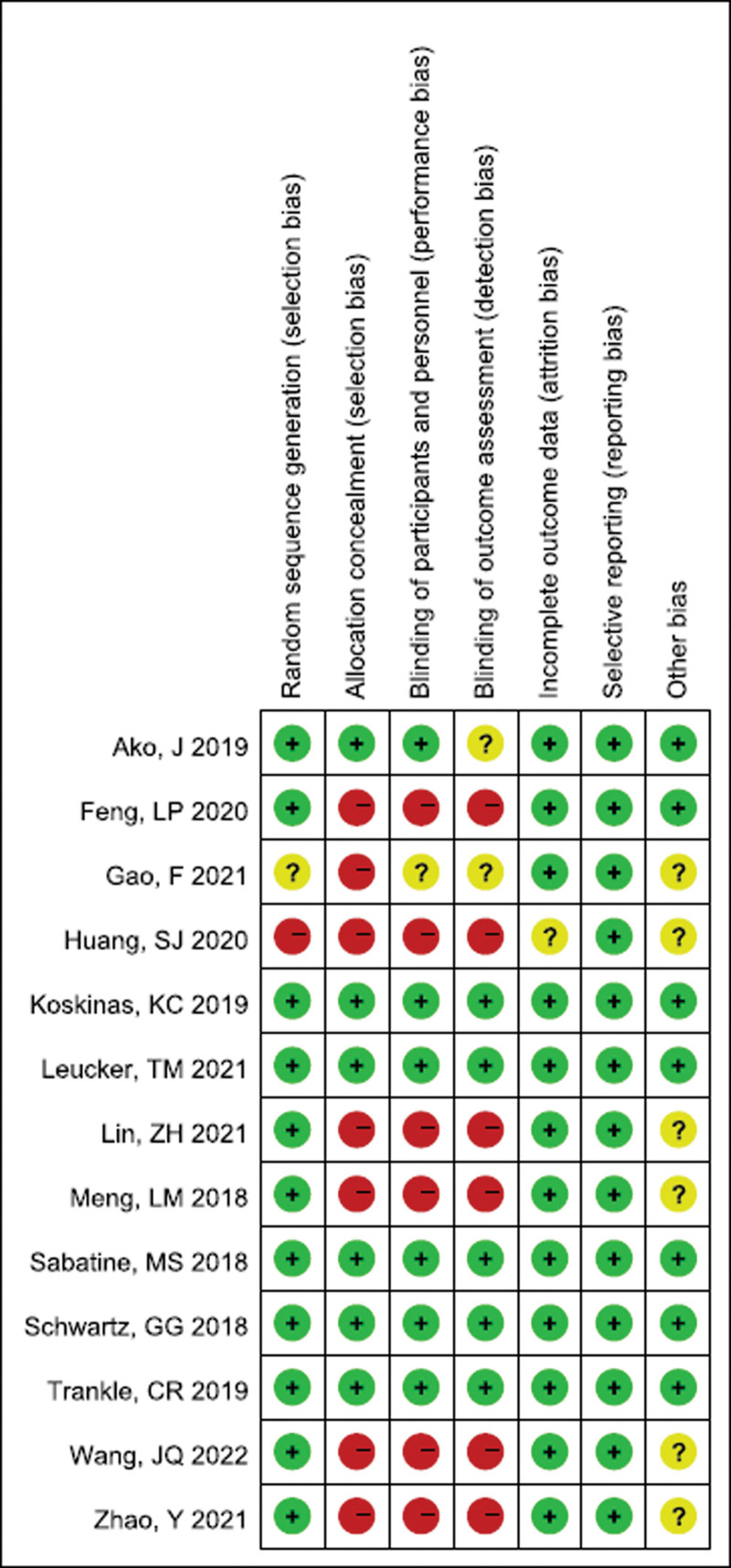
Results of quality assessment using Cochrane risk of bias tool for RCT. RCT = randomized controlled trial.

RCTs were assessed by the Cochrane risk of bias tool. The authors showed the results for each quality item as percentages across studies. One study did not report whether it was designed as an RCT, 1 study was ambiguous about its RCT design, and 11 studies clearly stated that they were designed as RCTs. Additionally, 7 studies did not report on allocation concealment, while 6 studies claimed to have implemented allocation concealment. Finally, 6 studies reported double-blind methods. The quality assessment of included studies is shown in Figure 2 for details. Retrospective studies were assessed by NOS. Although 1 study scored 6 on the NOS, 19 studies scores were ≥7, which were considered to be high quality. The detailed information can be found in Table 2.

**Table 2:** Results of quality assessment using Newcastle–Ottawa scale for cohort studies.

Study selection	Representativeness of the exposed cohort	Selection of the nonexposed cohort	Ascertainment of exposure	Demonstration of outcome of interest that did not exist at start of study	Comparability of cohorts on the basis of the design or analysis	Assessment of outcome	Follow-up long enough for outcomes to occur	Adequacy of follow-up of cohorts	Quality score
Ye et al^[28]^	1	1	1	1	1	1	1	1	8
Lei et al^[24]^	1	1	0	1	1	1	1	1	7
Tao et al^[25]^	1	1	1	1	1	0	1	0	6
Wang et al^[26]^	1	1	0	1	1	1	1	1	7
Li et al^[29]^	1	1	1	1	1	1	1	0	7
Xu et al^[30]^	1	1	1	1	1	1	1	1	8
Yano et al^[27]^	1	1	1	1	1	1	1	1	8

### 3.4. Meta-analysis results

#### 3.4.1. LDL-C levels

**Figure 3.**
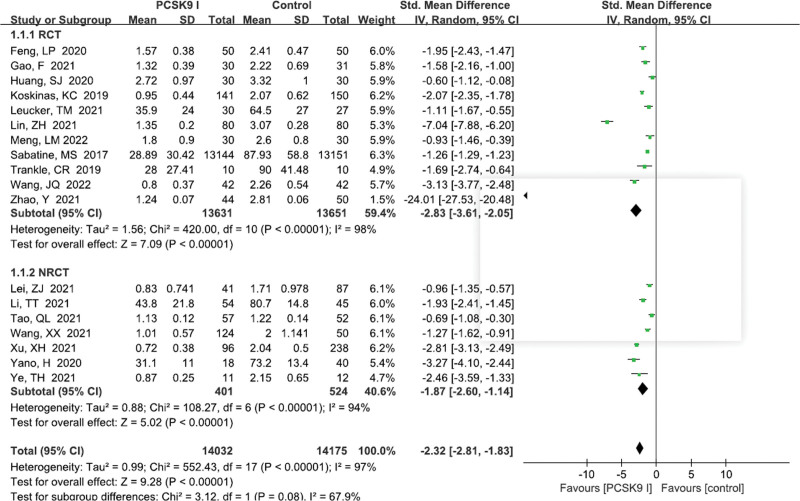
Forest plot showing LDL-C levels. CI = confidence interval, LDL-C = low density lipoprotein cholesterol, PCSK9 = proprotein convertase subtilisin kexin type, RCT = randomized controlled trial, SD = standard deviation.

A total of 18 studies^[11–16,18,20–30]^ reported the LDL-C levels. There existed a significant heterogeneity of studies (*P* < .00001, *I*^2^ = 97%). Random effects model was performed. Pooled results showed that PCSK9 inhibitors group was superior to control group (SMD = −2.32, 95% CI: −2.81 to −1.83, *P* < .00001, Fig. 3). What’s more, a subgroup analysis was conducted by the study design, intervention regimes of control group, and treatment dosage of PCSK9 inhibitor group. However, the subgroup analysis did not reveal the potential source of heterogeneity (Table 3).

**Table 3:** Subgroup analysis.

Stratification	No. of studies	No. of patients	Pooled SMD/MD	95% CI of pooled SMD/MD	*P* value	Heterogeneity *I *^2^, %
LDL-C level	18	28,207	−2.32	−2.81 to −1.83	<.00001	97
RCT	11	27,282	−2.83	−3.61 to −2.05	<.00001	98
NRCT	7	925	−1.87	−2.60 to −1.14	<.00001	94
Statins	15	1835	−2.75	−3.48 to −2.02	<.00001	97
Placebo	3	26,372	−1.26	−1.29 to −1.23	<.00001	0
75 mg q2w	2	84	−1.88	−2.70 to −1.06	<.00001	46
140 mg q2w	12	27,595	−2.13	−2.69 to −1.57	<.00001	97
150 mg 1 mo	1	20	−1.69	−2.74 to −0.64	.002	NA
420 mg every month	3	508	−3.37	−5.94 to −0.81	.01	99
HDL-C level	14	1741	0.17	−0.02 to 0.36	.08	70
RCT	7	816	0.23	−0.04 to 0.50	.10	71
NRCT	7	925	0.11	−0.18 to 0.40	.45	73
Statins	14	1741	0.17	−0.02 to 0.36	.08	70
Placebo	NA	NA	NA	NA	NA	NA
75 mg q2w	2	84	0.44	−0.16 to 1.04	.15	38
140 mg q2w	10	1206	0.19	−0.06 to 0.44	.14	75
420 mg every month	2	451	−0.03	−0.26 to 0.19	.78	29
TC level	12	1617	−1.24	−1.40 to −1.09	<.00001	74
RCT	7	849	−1.40	−1.55 to −1.24	<.00001	55
NRCT	5	768	−1.01	−1.33 to −0.70	<.00001	81
Statins	12	1617	−1.24	−1.40 to −1.09	<.00001	74
Placebo	NA	NA	NA	NA	NA	NA
75 mg q2w	1	23	−1.26	−1.79 to −0.73	<.00001	NA
140 mg q2w	9	1143	−1.20	−1.44 to −0.95	<.00001	80
420 mg every month	2	451	−1.35	−1.45 to −1.26	<.00001	0
TG level	14	1736	−0.36	−0.56 to −0.16	.0004	94
RCT	8	910	−0.51	−0.75 to −0.27	<.0001	94
NRCT	6	826	−0.15	−0.29 to −0.02	.03	49
Statins	14	1736	−0.36	−0.56 to −0.16	.0004	94
Placebo	NA	NA	NA	NA	NA	NA
75 mg q2w	2	84	−0.19	−0.63 to 0.26	.41	42
140 mg q2w	10	1201	−0.35	−0.56 to −0.14	.001	91
420 mg every month	2	451	−0.49	−1.24 to 0.25	.19	98

CI = confidence interval, MD = mean difference, SMD = standard mean difference.

#### 3.4.2. HDL-C levels

**Figure 4.**
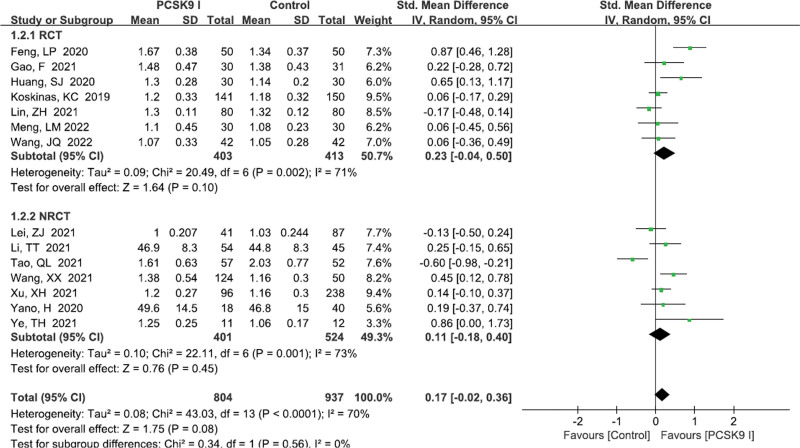
Forest plot showing HDL-C levels. CI = confidence interval, HDL-C = high density lipoprotein cholesterol, PCSK9 = proprotein convertase subtilisin kexin type, RCT = randomized controlled trial, SD = standard deviation.

A total of 14 studies^[11–14,16,18,23–30]^ reported the HDL-C levels. There existed a significant heterogeneity (*P* < .0001, *I*^2^ = 70%). Random effects model was performed. Pooled results showed that PCSK9 inhibitors group was not superior to control group (SMD = 0.17, 95% CI: −0.02 to 0.36, *P* = .08; Fig. 4). However, the subgroup analysis did not reveal the potential source of heterogeneity (Table 3).

#### 3.4.3. TC levels

**Figure 5.**
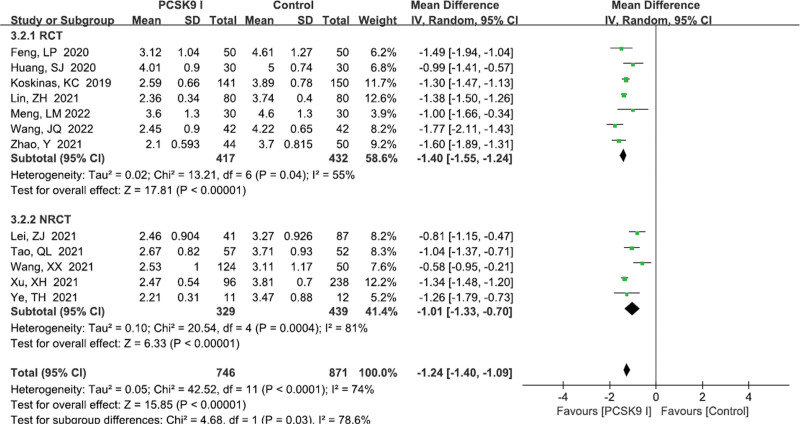
Forest plot showing TC levels. CI = confidence interval, PCSK9 = proprotein convertase subtilisin kexin type, RCT = randomized controlled trial, SD = standard deviation, TC = total cholesterol.

A total of 12 studies^[11–15,18,23–26,28,30]^ reported the TC levels. There was significant heterogeneity (*P* < .0001, *I*^2^ = 74%). Random effects model was performed. Pooled results showed that PCSK9 inhibitors group were superior to control group (MD = −1.24, 95% CI: −1.40 to −1.09, *P* < .00001; Fig. 5). However, the subgroup analysis did not reveal the potential source of heterogeneity (Table 3).

#### 3.4.4. TG levels

**Figure 6.**
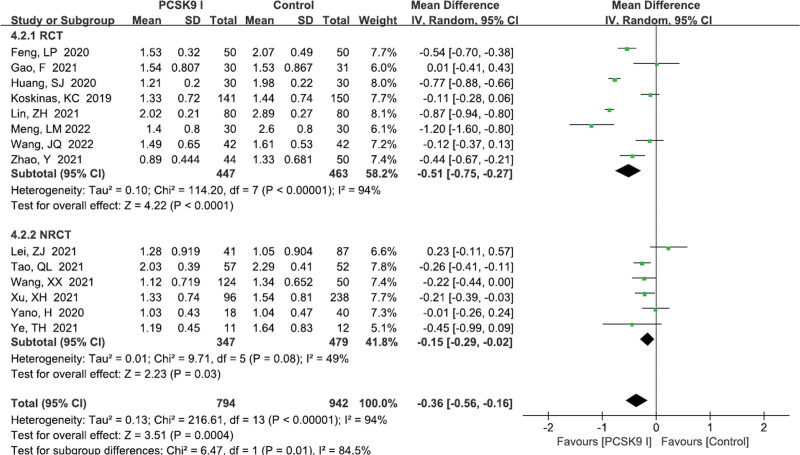
Forest plot showing TG levels. CI = confidence interval, PCSK9 = proprotein convertase subtilisin kexin type, RCT = randomized controlled trial, SD = standard deviation, TG = triglyceride.

A total of 14 studies^[11–16,18,23–28,30]^ reported TG levels. There exists significant heterogeneity (*P* < .00001, *I*^2^ = 94%). Random effects model was performed. Pooled results showed that PCSK9 inhibitors group were superior to control group (MD = −0.36, 95% CI: −0.56 to −0.16, *P* = .0004; Fig. 6). The subgroup analysis did not reveal the potential source of heterogeneity (Table 3).

### 3.5. Safety

#### 3.5.1. MACEs

**Figure 7.**
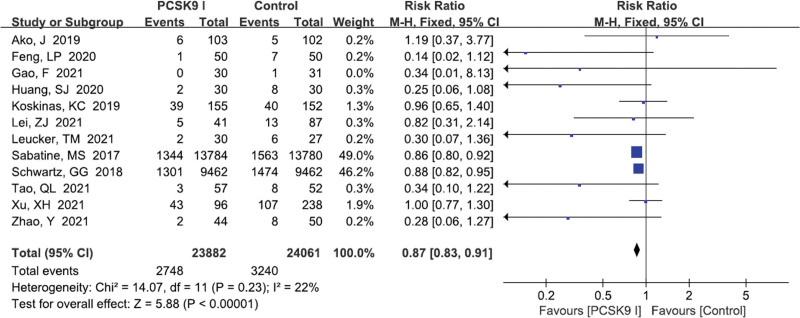
Forest plot showing MACEs. CI = confidence interval, MACEs = major adverse cardiovascular events, PCSK9 = proprotein convertase subtilisin kexin type.

Twelve studies^[12,13,15–19,21,22,24,25,30]^ reported the incidence of MACEs. The results demonstrated that there was statistical difference between the 2 groups (RR = 0.87, 95% CI: 0.83–0.91, *P* < .00001, Fig. 7). No significant heterogeneity (*I*^2^ = 22%, *P* = .23) was found, suggesting that patients used PCSK9 inhibitors were safer.

##### 3.5.1.1. Myocardial infarction

Ten studies^[12,15–19,22,24,25,30]^ reported the incidence of myocardial infarction. The results demonstrated that there was statistical difference between the 2 groups (RR = 0.80, 95% CI: 0.74–0.86, *P* < .00001, Table 4). No heterogeneity (*I*^2^ = 35%, *P* = .12) was found. The result demonstrated that patients used PCSK9 inhibitors could reduce myocardial infarction than those who used statins or placebo treatment.

**Table 4:** Main cardiovascular adverse events.

Stratification	No. of studies	No. of patients	Pooled RR	95% CI of pooled RR	*P* value	Heterogeneity *I *^2^, %
Myocardial infarction	10	47,826	0.80	0.74–0.86	<.00001	35
Angina	6	47,085	0.90	0.83–0.97	<.00001	22
Cardiac death	7	47,211	0.92	0.83–1.03	.16	0

CI = confidence interval, RR = relative risk.

##### 3.5.1.2. Angina

Six studies^[13,15,19,22,25,30]^ reported the incidence of angina. The results demonstrated that there was statistical difference between the 2 groups (RR = 0.90, 95% CI: 0.83–0.97, *P* = .008, Table 4). No significant heterogeneity (*I*^2^ = 22%, *P* = .27) was found. The result demonstrated that patients used PCSK9 inhibitors could reduce angina than those who used statins or placebo treatment.

##### 3.5.1.3. Cardiac death

Seven studies^[12,15,17–19,22,24]^ reported the incidence of cardiac death. The results demonstrated that there was no statistical difference between the 2 groups (RR = 0.92, 95% CI: 0.83–1.03, *P* = .16, Table 4). No significant heterogeneity (*I*^2^ = 0%, *P* = .42) was found. The result demonstrated that patients used PCSK9 inhibitors could not reduce cardiac death than those who used statins or placebo treatment.

#### 3.5.2. Drug-induced adverse events

**Figure 8.**
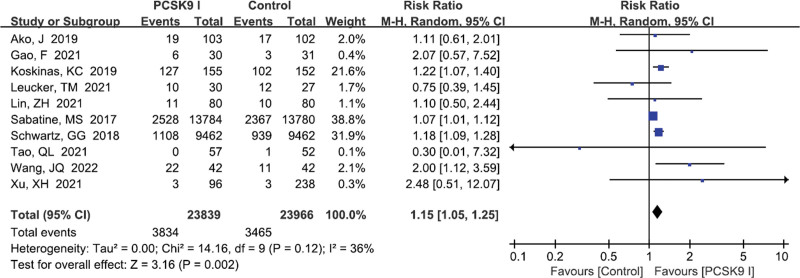
Forest plot showing Drug-induced adverse events. CI = confidence interval, PCSK9 = proprotein convertase subtilisin kexin type.

Ten studies^[14,16–19,21,22,24,25,30]^ reported the incidence of drug-induced adverse events. The results demonstrated that there was statistical difference between the 2 groups (RR = 1.15, 95% CI: 1.05–1.25, *P* = .002, Fig. 8). The result demonstrated that patients used PCSK9 inhibitors had a higher incidence of drug-induced adverse events than those who used statins or placebo treatment.

#### 3.5.3. Other outcomes

We also reported the other clinical outcomes included apolipoprotein B, LDL-C change from baseline, LDL-C <1.4, and Lipoprotein A. All results can be seen supplemental file, Supplemental Digital Content, http://links.lww.com/MD/M665.

### 3.6. Publication bias

**Figure 9.**
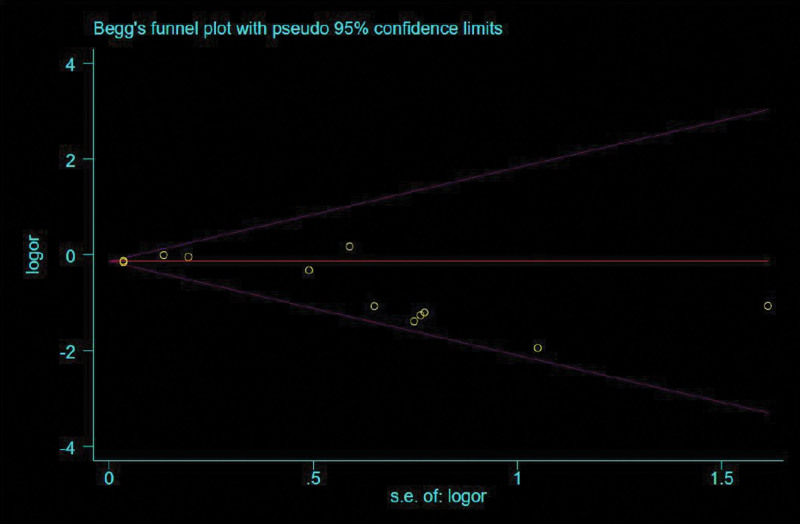
Begge funnel plot.

A funnel plot was used to evaluate the publication bias. The funnel plot was asymmetrical. This indicated the possibility of publication bias. And, when the Begg funnel plot test’s was performed (Fig. 9), the result of the evaluation indicates a possible existence of publication bias (*P* = .034).

## 4. Discussion

In recent years, PCSK9 inhibitors have emerged as a novel therapy capable of lowering blood lipids and modulating inflammation.^[31]^ Key RCTs, including the ODYSSEY long-term trail,^[32]^ FOURIER trial,^[22]^ and GLAGOV trial,^[33]^ have underscored the ability of PCSK9 inhibitors to reduce MACEs. Furthermore, these inhibitors have shown efficacy in lowering LDL-C levels.^[16,18,19]^ Despite these promising findings, there is currently a lack of systematic reviews and meta-analyses evaluating the safety and efficacy of PCSK9 inhibitors specifically for patients with ACS. Therefore, conducting a comprehensive meta-analysis on the use of PCSK9 inhibitors in ACS patients is imperative.

Elevated blood lipid levels can heighten the risk of cardiovascular complications. Effective management of lipid profiles, particularly LDL-C levels, plays a pivotal role in mitigating cardiovascular diseases and related events.^[34]^ Accurate assessment of LDL-C is essential for guiding cholesterol-lowering interventions.^[35]^ In the FOURIER trial, a randomized, double-blind, placebo-controlled, multinational clinical study, it was reported that after 48 weeks, treatment with PCSK9 inhibitors led to a substantial mean percentage reduction of 59% in LDL-C levels compared to the placebo group (*P* < .001), resulting in an average absolute reduction of 56 mg per deciliter (*P* < .001).^[22]^ Another large RCT from the ODYSSEY trial also arrived at a similar conclusion.^[32]^ By synthesizing the findings of these 18 studies, encompassing a total of 28,207 patients, this meta-analysis revealed that the use of PCSK9 inhibitors resulted in reduced LDL-C levels in patients with ACS compared to those receiving statins alone or placebo. However, it is important to note that there was considerable heterogeneity among the included studies, and therefore, the results should be interpreted with caution. Subgroup analysis indicated that this heterogeneity may be attributed to factors such as race, dosage and frequency of PCSK9 inhibitors, statin dosage, and duration of follow-up.

The decrease of HDL-C is a dyslipidemia that leads to atherosclerosis, reflecting metabolic impairment and increased cardiovascular risk. HDL-C is also considered to be a main means to preserve and possibly enhance cardiac protective function.^[36,37]^ And multiple factors through oxidation and inflammation might lead to HDL particle dysfunction or atherosclerosis.^[38,39]^ Most clinical trials have shown that PCSK9 inhibitors moderately increase HDL-C and apoA1 levels (usually 10%). A study on HDL-C also reported that the average change in PCSK9 inhibitors was 8% compared to placebo and 8% compared to ezetimibe (*P* > .05).^[40]^ PCSK9 gain-of-function mutations are associated with increased HDL-C levels and apoA1 levels.^[41]^

In the recent FOURIER trial, PCSK9 inhibitors were found to significantly elevate HDL-C levels by 8.4% and apoA1 levels by 6.5%, in contrast to minimal changes seen with placebo (0.3% and 2.1%, respectively; both *P* < .001).^[22,42]^ However, a meta-analysis of 18 studies concluded that the use of PCSK9 inhibitors did not demonstrate superiority over statins alone or placebo in patients with ACS. The authors suggested that the larger sample size and diverse participant backgrounds in this meta-analysis could yield different results compared to previous studies. Elevated TG and TC levels have been identified as risk factors for atherosclerotic cardiovascular disease, with potential implications for predicting cardiovascular outcomes in patients.^[43,44]^ Studies have indicated that the addition of PCSK9 inhibitors to statin therapy effectively reduces TC, TG, and LDL-C levels.^[45]^ Similarly, findings from the GLAGOV randomized clinical trial revealed that PCSK9 inhibitors significantly lowered TC (108.6 mg/dL) and TG levels (105.1 mg/dL) compared to placebo (169.1 and 130.5 mg/dL, respectively; both *P* < .001).^[33]^ These results suggest that PCSK9 inhibitors offer certain advantages in reducing LDL-C levels, TC levels, and TG levels in ACS patients compared to treatment with statins alone or placebo. Furthermore, it is noteworthy that the use of PCSK9 inhibitors has been associated with a significant reduction in long-term MACEs among patients with ACS.

Elevated levels of LDL-C play a crucial role in predicting the occurrence of ACS and MACEs. High LDL-C levels can result in the accumulation of cholesterol in the arterial walls, leading to the development of plaques. Over time, these plaques can narrow the coronary arteries, potentially causing plaque rupture and blood clot formation, which may lead to myocardial infarction and unstable angina. Furthermore, abnormal TC and TG levels are also linked to an increased risk of ACS and MACEs. By effectively managing blood lipid levels, particularly by reducing LDL-C, TC, and TG levels, it is possible to lower the incidence of MACEs in patients with ACS. Numerous large-scale randomized clinical trials have consistently shown a decrease in MACEs rates with the use of PCSK9 inhibitors in individuals with established cardiovascular disease. The ODYSSEY OUTCOMES trial suggested that the incidence of MACEs in the PCSK9 inhibitors group was lower than that in the control group (*P* < .001), and a total of 334 patients (3.5%) in the PCSK9 inhibitors group and 392 patients (4.1%) in the control group experienced MACEs, with no significant difference observed between the groups (*P* = .38). The incidence of drug-induced adverse events associated with PCSK9 inhibitors was higher compared to the control group (3.8% vs 2.1%, *P* < .001).^[22]^ Similar findings were reported in The FOURIER trial.^[46]^ In a meta-analysis of 20 studies, it was observed that the incidence of MACEs in the PCSK9 inhibitors group was lower than in those using statins alone or placebo. Furthermore, this meta-analysis revealed that PCSK9 inhibitors can effectively reduce the occurrence of myocardial infarction and angina when analyzed separately. However, there is a possibility that PCSK9 inhibitors may increase the risk of drug-induced adverse events. Previous reviews have suggested that PCSK9 inhibitors decrease the risk of MACEs compared to the control group, but do not reduce the risks of all-cause mortality. The research shows that the overall incidence of adverse reactions to statin drugs is 7.46%; among them, gastrointestinal symptoms account for 3.94%; liver symptoms account for 1.3%; muscle symptoms account for 1.17%; and neurological symptoms account for 0.65%. And one of the most commonly reported adverse reactions to PCSK9 inhibitors is local injection-site reactions, typically mild (erythema, pain, or bruising), with overall adverse event rates ranging from 7% to 10%.^[47,48]^ Moreover, this meta-analysis found that PCSK9 inhibitors had higher drug-induced adverse events compared to control group (RR = 1.15, 95% CI: 1.05–1.25, *P* = .002). Our study findings align with these previous reviews and stand out as the most comprehensive review due to our thorough evaluation of literature from various sources and inclusion of numerous large multicenter clinical studies.

This study has some limitations: some of the aggregated results included in the study are subjective, which may affect the results due to different experiences of doctors. The follow-up time of each study is different, and the follow-up time of trials is usually short, so the long-term effects of PCSK9 inhibitors (including benefits and harms) are still unknown. The heterogeneity of continuous variables is high, so meta-analysis showed low quality, and we should further analyze the source of heterogeneity. Three articles were not available when we included relevant literatures, so this added a certain bias. Therefore, physicians around the world should interpret our results with cautions when applying them in clinical practice.

## 5. Conclusion

Although this study demonstrates that PCSK9 inhibitors have higher drug-induced adverse events, they can not only reduce LDL-C levels but also reduce MACEs simultaneously. However, these findings needed to be further verified through large sample, multicenter, double-blind RCTs.

## Author contributions

**Conceptualization:** Ruohong Song, Xiaojian Liu, Qiyong Li.

**Data curation:** Ruohong Song, Qiyong Li.

**Formal analysis:** Ruohong Song.

**Writing—original draft:** Ruohong Song, Yan Xiong, Hui Huang, Xiaojian Liu, Qiyong Li.

**Investigation:** Jinsong Li.

**Methodology:** Jinsong Li.

**Writing—review & editing:** Jinsong Li, Xiaojian Liu, Qiyong Li.

**Project administration:** Yan Xiong.

**Resources:** Yan Xiong.**Software:** Yan Xiong.

**Supervision:** Hui Huang.

**Validation:** Hui Huang.

**Visualization:** Hui Huang.

## Supplementary Material


