# Supplementary material for: Efficacy and safety of proprotein convertase subtilisin kexin type (PCSK9) inhibitors in patients with acute coronary syndrome: A systematic review and meta-analysis
Source: Medicine (Baltimore). 2024 May 31;103(22):e38360. doi: 10.1097/MD.0000000000038360 (PMC11142774; doi:10.1097/MD.0000000000038360)
Supplement: Supplementary file 1 [file medi-103-e38360-s001.docx]

Other outcomes of the meta-analysis

| Stratification | No. of studies | No. of patients | Pooled SMD/MD/RR | 95% CI | P value | Heterogeneity I^2^ (%) |
| --- | --- | --- | --- | --- | --- | --- |
| ApoB | 4 | 648 | -1.73 | -2.29 – -1.17 | 0.00001 | 88 |
| LDL-C change from baseline | 7 | 1221 | 3.87 | 2.54 – 5.21 | 0.00001 | 98 |
| LDL-C<1.4 | 6 | 875 | 24.68 | 7.90-77.12 | 0.00001 | 84 |
| Lipoprotein A | 5 | 742 | 0.09 | -0.27-0.46 | 0.62 | 82 |

ApoB=apolipoprotein B; LDL-C=low density lipoprotein cholesterol; MD=mean difference; SMD=standard mean difference; RR=relative risk; CI=confidence interval.
